# Outcomes of stable and unstable patterns of subjective cognitive decline – results from the Leipzig Longitudinal Study of the Aged (LEILA75+)

**DOI:** 10.1186/s12877-016-0353-8

**Published:** 2016-11-04

**Authors:** Susanne Roehr, Arno Villringer, Matthias C. Angermeyer, Tobias Luck, Steffi G. Riedel-Heller

**Affiliations:** 1 Institute of Social Medicine, Occupational Health and Public Health (ISAP), University of Leipzig, Leipzig, Germany; 2 Max Planck Institute for Human Cognitive and Brain Sciences, Leipzig, Germany; 3 University Hospital Leipzig, Day Clinic for Cognitive Neurology, Leipzig, Germany; 4 Center for Public Mental Health, Gösing a. W., Austria; 5 Department of Public Health, Clinical and Molecular Medicine, University of Cagliari, Cagliari, Italy; 6 LIFE – Leipzig Research Center for Civilization Diseases, University of Leipzig, Leipzig, Germany

**Keywords:** Subjective cognitive decline, Mild cognitive impairment, Dementia, Alzheimer’s disease, Progression risk, Cohort studies, Outcomes

## Abstract

**Background:**

Subjective cognitive decline (SCD), i.e., the self-perceived feeling of worsening cognitive function, may be the first notable syndrome of preclinical Alzheimer’s disease and other dementias. However, not all individuals with SCD progress. Stability of SCD, i.e., repeated reports of SCD, could contribute to identify individuals at risk, as stable SCD may more likely reflect the continuous neurodegenerative process of Alzheimer’s and other dementias.

**Methods:**

Cox regression analyses were used to assess the association between stability of SCD and progression to MCI and dementia in data derived from the population-based Leipzig Longitudinal Study of the Aged (LEILA75+).

**Results:**

Of 453 cognitively unimpaired individuals with a mean age of 80.5 years (*SD* = 4.2), 139 (30.7 %) reported SCD at baseline. Over the study period (*M* = 4.8 years, *SD* = 2.2), 84 (18.5 %) individuals had stable SCD, 195 (43.1 %) unstable SCD and 174 (38.4 %) never reported SCD. Stable SCD was associated with increased risk of progression to MCI and dementia (unadjusted HR = 1.8, 95 % CI = 1.2–2.6; *p* < .01), whereas unstable SCD yielded a decreased progression risk (unadjusted HR = 0.5, 95 % CI = 0.4–0.7; *p* < .001) compared to no SCD. When adjusted for baseline cognitive functioning, progression risk in individuals with stable SCD was significantly increased in comparison to individuals with unstable SCD, but not compared to individuals without SCD.

**Conclusions:**

Our results, though preliminary, suggest that stable SCD, i.e., repeated reports of SCD, may yield an increased risk of progression to MCI and dementia compared to unstable SCD. Baseline cognitive scores, though within a normal range, seem to be a driver of progression in stable SCD. Future research is warranted to investigate whether stability could hold as a SCD research feature.

## Background

Individuals with subjective cognitive decline (SCD), i.e., a self-perceived feeling of worsening cognitive function that is not objectifiable from neuropsychological testing, may have an increased risk to develop mild cognitive impairment (MCI) and dementia [1, 2]. In fact, SCD may be the first notable syndrome of preclinical Alzheimer’s disease (AD) and other dementias [3]. In studies with more than four years observation time, it was 26.6 % of those with SCD who developed MCI, and 14.1 % who developed dementia, as a meta-analysis revealed [1]. This cumulated in a doubled risk of dementia compared to cognitively unimpaired elderly without SCD. Thus, SCD could hold value for the early detection of future cognitive decline and, subsequently, as a starting point for early prevention and intervention trials. This would be particularly interesting as at the SCD stage, global cognitive functioning may be well preserved.

However, the prognosis from SCD is unclear precisely because not all individuals with SCD progress to MCI or dementia. In fact, SCD is a frequent syndrome prevalent in 25 to 50 % of the elderly [4], and besides being a risk factor for MCI and dementia, SCD could also be a) a functional manifestation of various underlying causes (e.g., depression, medication, personality traits) [3, 5], or b) a distorted awareness of normal performance as impaired [6]. Consequently, it would be useful to be able to better differentiate between individuals with SCD who progress to MCI and dementia from individuals with SCD who do not progress.

One such approach may lay in the investigation of the stability of SCD. Previous studies on MCI and dementia progression usually relied on a single measurement point of SCD leaving stability unaddressed [1, 2]. Moreover, due to the varying possible underlying causes, such subjectively perceived cognitive impairment in general is considered a quite unstable syndrome, and it can revert to a subjectively unimpaired experience of cognitive functioning instead of progressing to MCI or dementia [7, 8]. Reisberg et al. [9] stated, there is a need to clarify whether “SCD inexorably progresses into MCI and ultimately AD”, or whether “it is inherently less stable, varying with yet undermined factors (microvascular disease and/or mood)”.

The typical course of late onset Alzheimer’s disease (AD), the most frequent type of dementia, is marked by a long continuous process of pathological brain changes which evolve years before diagnosis [10, 11]. If SCD occurs due to preclinical AD, we could assume that SCD might be experienced rather constantly over time, hence, being a more stable syndrome that is reported repeatedly – as opposed to SCD due to other underlying causes that might lead to a more unstable experience of SCD, e.g., due to mood.

### Aims of the study

We aimed to longitudinally investigate multiple time points of SCD and its stability in regard to progression to MCI and dementia in a population-based sample of cognitively unimpaired elderly (≥75 years) who were followed over 8 years in total.

## Methods

### Study design and sample

Data were derived from the Leipzig Longitudinal Study of the Aged (LEILA75+), a population-based study on the epidemiology of dementia and MCI. Initially, a total of 1692 individuals aged at least 75 years residing in the Leipzig-South district were selected for participation. Of these, 1500 individuals were identified by systematic random sampling from an age-ordered list from the local registry office. In addition, 162 institutionalized individuals were included by systematic random sampling from an age-ordered list by the four institutions in the study area. Study details have been published elsewhere [12].

Of the 1692 invited individuals, 242 (14.2 %) refused, 57 (3.4 %) had died, 15 (0.9 %) were not traceable, and 113 (6.7 %) were shielded by relatives. Finally, the LEILA75+ cohort comprised 1265 (74.8 %) individuals. Non-participants did not differ from participants regarding age (*U =* 263553, *p =* .46), gender (*χ* ^*2*^ = 0.40, *p =* .53), or marital status (*χ* ^*2*^ = 5.03, *p =* .17).

### Data collection

Data were collected over a total observation period of eight years between January 1997 (begin of baseline) and April 2005 (end of follow-up 5). Follow-up assessments took place on average every 1.4 years. Structured clinical interviews at baseline and follow-up were conducted at participants’ homes by trained psychologists and physicians. In addition, structured interviews were held with proxies.

### Assessment instruments and procedures

The main assessment instrument was the Structured Interview for the Diagnosis of Dementia of Alzheimer type, Multi-infarct Dementia and Dementia of other Aetiology according to DSM-III-R, DSM-IV, and ICD-10 (SIDAM) [13]. The SIDAM comprises a neuropsychological test and a section for clinical judgment and third-party information on psychosocial impairment, including a 14-item scale to assess activities of daily living (SIDAM-ADL Scale). The SIDAM neuropsychological test consists of 55 items, including all 30 items of the Mini-Mental State Examination (MMSE) [14]. Six areas of neuropsychological functioning are covered: 1) orientation (time and place); 2) memory (delayed verbal recall, delayed visual reproduction, questions on biography and history); 3) intellectual abilities (abstract thinking) and judgment (plausibility judgments, describing pictures representing actions); 4) verbal and calculation abilities (calculating serial sevens, spelling backward, backward digit span); 5) visual-spatial constructional abilities (copying figures); and 6) aphasia and apraxia (naming objects, reading and obeying a sentence, writing a sentence, and performing a three-stage command).

If it was not possible to administer the SIDAM, a structured proxy interview was offered including the Clinical Dementia Rating scale (CDR) [15].

SCD was evaluated prior to cognitive testing by asking the participant: “Do you have problems with your memory?”.

We identified depressive symptoms using the German version of the 20-item Center of Epidemiologic Studies Depression Scale (CES-D) [16].

A standardized interview provided information on sociodemographic characteristics.

Death dates were obtained from relatives or the registry office.

### Definition of cases

#### SCD

SCD was assumed if participants were cognitively unimpaired and stated to have memory problems unrelated to an event or condition explaining the memory problems according to recent research criteria [3]. Consequently, we excluded participants who met the following criteria: 1) a diagnosis of MCI or dementia, 2) a MMSE score below 26 points, 3) presence of a major psychiatric (e.g., major depression, anxiety), neurological (e.g., Morbus Parkinson) or medical condition (e.g., stroke) that could affect cognitive functioning.

We then built two subgroups of SCD: stable vs. unstable SCD. Stable SCD was assumed if SCD was consistently reported at every assessment until progression to MCI or dementia or, in case of non-progression, the last completed follow-up. By contrast, unstable SCD was assumed if SCD was not consistently reported at every assessment, but occasionally, until progression to MCI and dementia or, in case of non-progression, the last completed follow-up.

#### Controls

Individuals who never reported SCD at baseline and follow-up until progression to MCI or dementia or last completed follow-up without progression were considered controls (CON).

#### MCI

Diagnosis of MCI was based on Winblad criteria [17]. They comprised absence of dementia, at most minimal impairment in instrumental activities of daily living, and evidence of cognitive decline in objective cognitive tests at least one standard deviation below age- and education specific norms [18] on one or more main domain of cognitive functioning as assessed by the SIDAM. We refrained from the criterion of the presence of a memory complaint to be able to consider any case of cognitive impairment in differentiation to CON and SCD [19].

#### Dementia

Dementia status at any assessment was agreed at consensus conferences with physicians and psychologists according to DSM-IV criteria [20]. The diagnosis was based on SIDAM results or, if proxy interviews only, on CDR data.

#### Outcome

We dichotomously defined the outcome variable in progression (to MCI or dementia) and non-progression. Progression was assigned if participants were first-time diagnosed with incident MCI or dementia at follow-up. Non-progression was assumed if individuals had remained CON or SCD at every completed follow-up.

### Statistical analysis

Group differences in socio-demographic and health characteristics at baseline between individuals with stable vs. unstable patterns of SCD and CON were analyzed applying Kruskal-Wallis-tests for continuous variables and *χ* ^*2*^ tests for categorical variables.

We developed univariate (model 1) and multivariate Cox proportional hazards models (models 2–4) to assess the association of progression to MCI and dementia in stable vs. unstable patterns of SCD in reference to CON.

Multivariate Cox models were stepwise adjusted for age, gender, education (categorized into low, middle and high according to the CASMIN criteria [21], categorical) (model 2), depressive symptoms (continuous, CES-D) [16]) (model 3), and cognitive functioning (continuous, MMSE [14]) (model 4).

Kaplan-Meier survival analyses were applied to determine time to progression in regard to SCD stability. Time to progression was defined as the interval from baseline to first follow-up of progression to MCI or dementia or, in case of non-progression, date of last completed follow-up. Individuals without progression by follow-up 5 were treated as censored data. A Log-rank test was performed to assess the unadjusted difference in time to progression between individuals with stable vs. unstable patterns of SCD vs. CON. Furthermore, a stratified Cox regression-based test for equality of survival was performed adjusted for age, gender, and education.

For all analyses a significance level of *α* = 0.05 was applied. We used Stata/SE, version 13.0 (StataCorp LP, College Station, Texas/USA).

## Results

### Participants

**Fig. 1:**
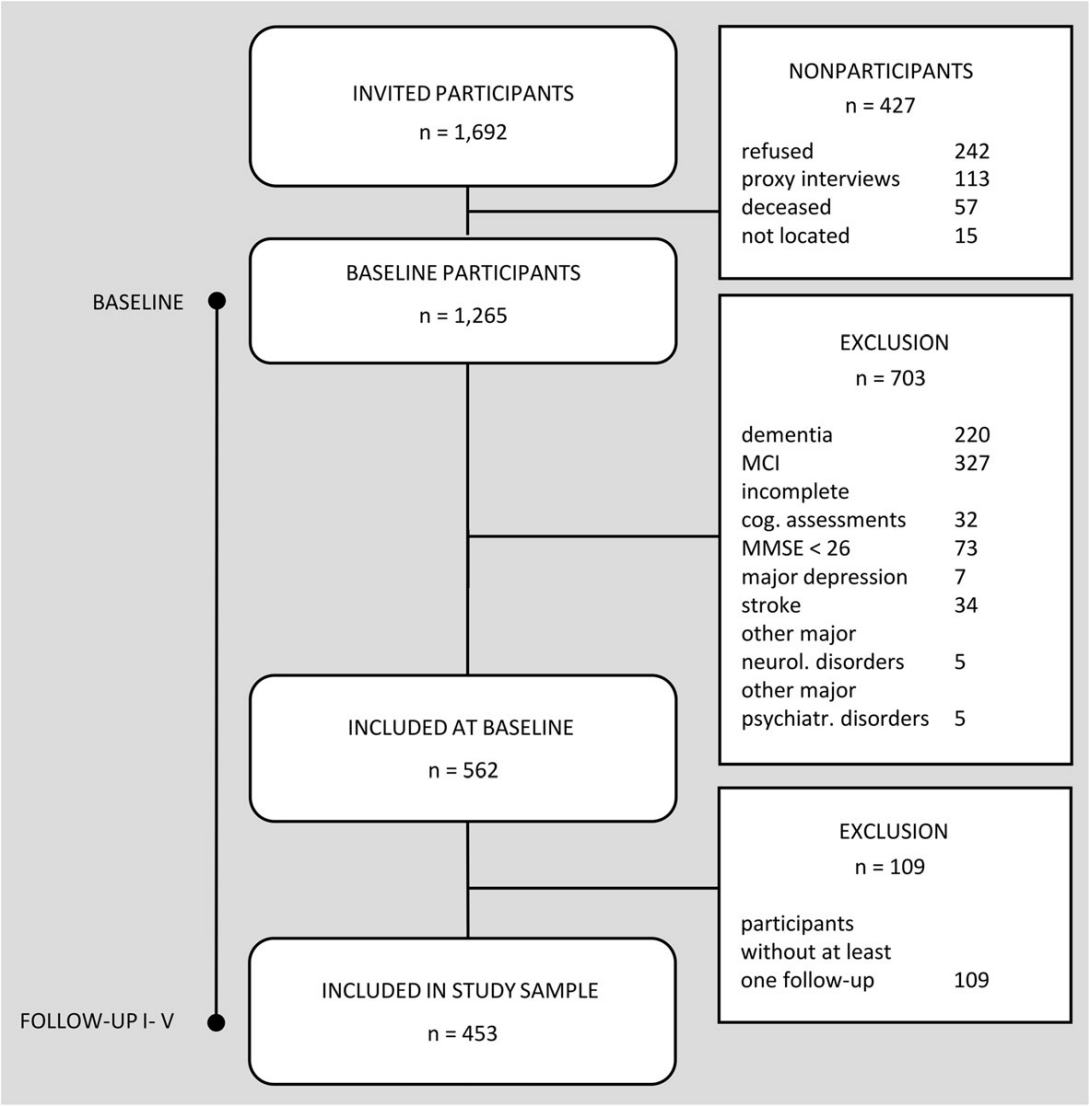
Sample and sample attrition

Of the 1265 LEILA75+ participants at baseline, 812 (64.2 %) individuals were excluded, primarily because of dementia (*n* = 220; 17.4 %), MCI (*n* = 327; 25.8 %), MMSE < 26 points (*n* = 73; 5.8 %), incomplete cognitive assessment (*n* = 32; 2.5 %), major depression according to DSM-IV criteria (*n* = 7; 0.6 %), stroke (*n* = 34; 2.7 %), other major psychiatric diseases (*n* = 5; 0.4 %), substance abuse (*n* = 5; 0.4 %), and not completing at least one follow-up (*n* = 109; 8.6 %) (Fig. 1). The final analysis pool comprised 453 (35.8 %) individuals.

### Characteristics of the study sample

Among the 453 study participants, 139 (30.7 %) individuals reported SCD at baseline. Over the study period, 84 (18.5 %) had stable SCD, 195 (43.0 %) had unstable SCD and 174 (38.4 %) never reported SCD (CON). Individuals with stable patterns reported SCD at 3 consecutive assessments (every 1.4 years) on average (*M* = 2.7; *SD* = 1.4). In those individuals with unstable SCD, reporting of SCD and no SCD switched twice on average (*M* = 1.7; *SD* = 0.8) between assessments. Individuals in the CON group never reported SCD over *M* = 3.1 (*SD* = 1.8) assessments.

The mean age of the participants was 80.5 years (*SD* = 4.2), of them 328 (72.7 %) were women. Individuals with stable SCD were significantly older, had slightly lower MMSE scores, and higher depressive symptoms than individuals with unstable SCD and CON (Table 1). There were no group differences regarding gender and education.

**Table 1:** Characteristics of the study sample at baseline (*n* = 453)

Variables^a^	Total (*n* = 453)	Stable SCD (*n* = 84)	Unstable SCD (*n* = 195)	CON (*n* = 174)	*P* Value (group difference)^b^
Age, mean (*SD*)	80.49 (4.21)	81.65 (4.49)	80.20 (4.07)	80.01 (4.06)	<.05
Gender, *n* (*%*)					
Male	125 (27.6)	24 (28.6)	58 (29.7)	43 (24.7)	
Female	328 (72.7)	60 (71.4)	137 (70.3)	131 (75.3)	.55
Education, *n* (*%*)					
Low	306 (67.7)	59 (71.1)	140 (71.8)	107 (61.5)	
Middle	92 (20.4)	13 (15.7)	35 (17.9)	44 (25.3)	
High	54 (11.9)	11 (13.3)	20 (10.3)	23 (13.2)	.20
Cognitive functioning/MMSE, mean (*SD*)	28.14 (1.27)	27.76 (1.19)	28.19 (1.31)	28.27 (1.23)	<.01
Depressive symptoms/CES-D, mean (*SD*)	13.55 (7.25)	16.47 (7.17)	13.64 (6.99)	12.07 (7.20)	<.001
Follow-up time, years, mean (*SD*)	4.83 (2.22)	4.01 (2.11)	5.61 (1.90)	4.37 (2.32)	<.001

*Abbreviations*: *CES-D* 20-item Center of Epidemiologic Studies Depression Scale, *CON* controls/individuals without SCD, *MMSE* Mini-Mental State Examination, *SCD* subjective cognitive decline

^a^Missing values, *n*(%): education = 1 (0.2 %); depressive symptoms: 28 (6.2 %); group statistic based on Kruskal-Wallis-tests for continuous variables and χ2 tests categorical variables

^b^Stable SCD vs. unstable SCD vs. CON

The total follow-up time was 8 years, the mean follow-up time cumulated in 4.8 years (*SD* = 2.2). Almost half of all participants (*n* = 218; 48.1 %) completed all assessments waves. Of 235 (51.9 %) individuals who were lost to follow-up, 122 (26.9 %) had deceased, 94 (20.7 %) refused further participation, and in 19 (4.2 %) contacting failed or they were lost for other reasons.

Individuals who were lost to follow-up were significantly older (*M* = 81.4, *SD* = 4.5 vs. *M* = 79.5, *SD* = 3.7; *p* < .001), more frequently male (*χ* ^*2*^(1, 453) = 4.56, *p* < .05) and had lower MMSE scores (*M* = 28.0, *SD* = 1.3 vs. *M* = 28.3; *SD* = 1.2, *p* < .01), but did not differ in regard to education (*χ* ^*2*^(2, 453) = 3.20; *p* = .20).

### Stability of SCD and progression risk

During an average observation of *M* = 4.8 years (*SD* = 2.2), 49 (28.2 %) of 174 CON, 39 (20 %) out of 195 individuals with unstable SCD, and 36 (42.9 %) out of 84 individuals with stable SCD incidentally developed either MCI or dementia (*χ* ^*2*^(2, 453) = 16.49, *p* < .01). Unadjusted Cox regression revealed a significantly increased hazard ratio (HR) of 1.8 for progression to MCI and dementia in individuals with stable SCD in reference to CON (95 % confidence intervaI/CI = 1.2–2.6; *p* < .01) (Table 2). This association remained significant in the multivariate Cox model adjusted for age, gender, education (HR = 1.8, 95 % CI = 1.2–2.6; *p* < .05) and depressive symptoms (HR = 1.6, 95 % CI = 1.0–2.3; *p* < .05). However, when additionally controlled for cognitive functioning, HR of progression to MCI and dementia in individuals with stable SCD reduced to 1.4 (95 %-CI = 0.9–2.1) in reference to CON, which was not significant anymore (*p* = .13). Unstable SCD, by contrast, yielded a significantly lower risk of progression in the univariate as well as in all multivariate Cox models in reference to CON (Table 2). Furthermore, Wald tests to contrast between the stable and unstable SCD group indicated a significant difference in the progression risk in all four models (model 1: *χ* ^*2*^ = 38.22, *p* < .001; model 2: *χ* ^*2*^ = 32.94; *p* < .001; model 3: *χ* ^*2*^ = 30.27, *p* < .001; model 4: *χ* ^*2*^ = 28.67, *p* < .001).

**Table 2:** Unadjusted and adjusted hazard ratios (HR) of progression to mild cognitive impairment (MCI) and dementia by stable vs. unstable subjective cognitive decline (SCD) in reference to controls (no SCD)

		Model 1		Model 2		Model 3		Model 4	
		HR (95 %-CI)	*p*	HR (95 %-CI)	*p*	HR (95 %-CI)	*p*	HR (95 %-CI)	*p*
SCD	Reference: no SCD	1		1		1		1	
	Unstable SCD	0.53 (0.37–0.74)	<.001	0.56 (0.39–0.79)	<.01	.50 (0.35–0.72)	<.001	0.47 (0.33–0.69)	<.001
	Stable SCD	1.78 (1.24–2.55)	<.01	1.76 (1.20–2.58)	<.05	1.55 (1.04–2.32)	< .05	1.38 (0.91–2.09)	.13
Age		–		1.01 (1.01–1.02)	<.001	1.01 (1.01–1.02)	< .001	1.01 (1.00–1.02)	<.01
Gender	Reference: male	–		0.95 (0.67–1.34)	.76	0.96 (0.68–1.36)	.83	0.94 (0.67–1.34)	.75
Education	Reference: low	–		1		1		1	
	Middle			1.72 (1.21–2.44)	<.01	1.57 (1.09–2.27)	<.05	1.61 (1.11–2.33)	<.05
	High			1.36 (0.88–2.10)	.17	1.34 (0.85–2.11)	.20	1.54 (0.96–2.47)	.07
Depressive symptoms/CES-D		–		–		1.00 (1.00–1.01)	.05	1.00 (1.00–1.01)	.09
Cognitive functioning/MMSE		–		–		–		0.97 (0.94–0.99)	<.05

*Abbreviations*: CES-D 20-item Center of Epidemiologic Studies Depression Scale, *CI* confidence interval, *HR* hazard ratio, *MCI* mild cognitive impairment, *MMSE* Mini-Mental State Examination, *SCD* subjective cognitive decline

### Stability of SCD and time to progression

**Fig. 2:**
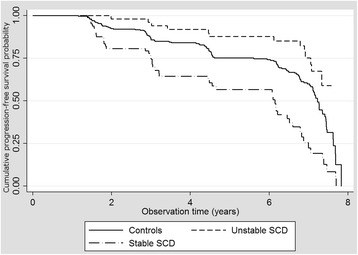
Kaplan-Meier cumulative probability of progression to mild cognitive impairment or dementia in stable vs. unstable subjective cognitive decline (SCD) vs. controls

The overall time to progression cumulated in 7.2 (95 % CI = 7.0–7.3) years in median as estimated by the Kaplan Meier Method (Table 3). Time to progression was significantly shorter in stable SCD (Median = 6.2 years, 95 % CI = 4.6–7.7) compared to unstable SCD (Median = 7.4, 95 % CI = 7.2–7.6) and CON (Median = 7.2, 95 % CI = 6.8–7.6; *p* < .001) (Fig. 2). When adjusted for age, gender and education, the difference in time to progression remained significant as estimated with a stratified Cox regression-based test for equality of survival (*χ* ^*2*^(2, 453) = 4.97, *p* < .05).

**Table 3:** Outcomes over the study period^a^ in individuals with stable vs. unstable patterns of subjective cognitive decline (SCD) and controls (CON)^b^

	Total (*n* = 453)	Stable SCD (*n* = 84)	Unstable SCD (*n* = 195)	CON (*n* = 174)	*P* Value (group difference)
No cognitive decline, *n* (%)	329 (59.4)	48 (57.1)	156 (80.0)	125 (71.8)	
Mild cognitive impairment (MCI), *n* (%)	69 (15.2)	21 (25.0)	19 (9.7)	29 (16.7)	
Dementia, *n* (%)	55 (12.1)	15 (17.9)	20 (10.3)	20 (11.5)	<.01
Time to cognitive decline, median (95 %-CI)	7.15 (7.01–7.29)	6.15 (4.59–7.70)	7.35 (7.15–7.55)	7.18 (6.79–7.56)	<.001

^a^total observation time: 8 years, mean observation time: 4.8 years (*SD* = 2.2)

^b^individuals without objective cognitive impairment and without SCD

## Discussion

We aimed to investigate stable vs. unstable patterns of subjective cognitive decline (SCD) in regard to progression to mild cognitive impairment (MCI) and dementia in a population-based sample of cognitively unimpaired elderly. Stable SCD, i.e., repeated reports of SCD, yielded an increased risk of progression to MCI and dementia compared to unstable SCD over an average observation period of 4.8 years.

Risk of progression seemed to be driven in part by baseline cognitive functioning in the stable SCD group, but not in the unstable SCD group.

There is a lack of studies that investigated stability of SCD in relation to MCI and dementia progression. One study, based on a memory clinic sample of 230 cognitively normal individuals with SCD over 50 years of age, distinguished three outcomes after eight years, namely non-decline, decline and diagnostically unstable (i.e., a change of diagnosis over time from normal to MCI, then back to normal) [22]. The authors concluded that the presence of SCD was a predictor of future cognitive decline, but it also increased the likelihood of an unstable diagnosis. Such an unstable diagnosis was associated with affective symptoms, particularly anxiety. The authors noted that the associations found in previous studies on cognitive decline and affective symptoms might be explained at least in part by including individuals with an unstable diagnosis who might later revert to normal. Besides, the possibility of reversion to “normal”, i.e., the perception of sufficient memory function with no more experience of SCD, seems much neglected in research as the reversion to normal cognition in MCI [23]. This may suggest, in line with our results, that it might be useful to distinguish between stable and unstable – including reversing – courses of SCD as they might relate to different aetiologies.

That stable SCD might be associated with an increased progression risk to MCI and dementia makes sense in the view of the fact that AD, the most frequent type of dementia, is characterized by a long continuous process of slow neurodegeneration into marked cognitive decline at advanced stages. It seems reasonable to assume that SCD, if a syndrome of preclinical AD, should be constantly experienced over a longer time period in this ongoing process rather than occasionally. Interestingly, this is underlined by our result that an unstable pattern of SCD, by contrast, was not predictive of MCI and dementia. In fact, individuals who did not consistently but only occasionally report SCD throughout the assessments displayed a decreased risk of MCI and dementia compared to individuals with stable SCD and those who never reported SCD. Potentially, this might be explained by temporary clinical conditions out of which SCD might result, e.g., due to a depressive episode, pain, fatigue, medications, or distressing life events [24]. As soon as such temporary conditions resolve, cognitive functioning may not be experienced as impaired anymore.

Otherwise, it has been reported previously that individuals in general struggle with differentiating between a normal and a pathological process of ageing [6] which could also be a reason for inconsistent reports of SCD. From a functional perspective, occasional reports of SCD might also be explained as a “distorted awareness of a present cognitive state compared to a past one” resulting in complaints of inexistent cognitive decline [6]. Individuals may over-estimate subtle changes in cognitive functioning that may only reflect age-related normative cognitive decline without becoming overt dementia later [25]. Over-estimation of cognitive changes could be driven by a fear of anticipatory dementia [26]. Such a fear is also associated with symptom-seeking for the disease [27].

By contrast, if SCD is completely absent, it could reflect either that there are indeed no memory problems or it could indicate poor awareness, i.e., the inability to accurately appraise aspects of cognitive functioning, also referred to as anosognosia [25]. If some of the individuals who did not express SCD had a poor awareness of cognitive changes, and thus, failed to express SCD, then this could explain why individuals who never reported SCD had an intermediate level of progression risk between the stable and unstable SCD group in our study. As there is evidence for variability in the level of awareness in MCI, it is likely to assume different levels of awareness in SCD where changes in cognitive functioning may be more subtle and, therefore, may be even more difficult to evaluate accurately [28]. However, whether differences in awareness are associated with progression risk to MCI and dementia is not known [25, 28].

As hypothesized, individuals with stable SCD exhibited the highest progression risk to MCI and dementia compared to individuals with unstable SCD and no SCD. When adjusted for cognitive functioning (MMSE scores), however, progression risk in individuals with stable SCD was only significantly increased in comparison to individuals with unstable SCD, but not compared to individuals without SCD. We assume, that the averagely slightly lower MMSE scores in individuals with stable SCD could rather reflect early pre-clinical cognitive decline as opposed to in individuals with unstable SCD. It has been previously reported, e.g., by St John and Montgomery [29], that individuals with SCD (regardless of its stability) experience “real” cognitive losses, “which is also apparent as lower [cognitive] scores”. Reisberg et al. [9] added that a lower MMSE score in individuals with SCD is not simply statistically significant, but it may be “real” in that individuals with SCD experience pre-clinical cognitive losses. Our results additionally suggest that this may particularly apply to individuals who have a stable experience of SCD as opposed to an unstable experience of SCD.

As the need for a refinement of the research criteria of SCD has been pointed out [3, 7], our preliminary results suggest that stability of SCD might be a feature that could contribute to better identify individuals at risk for MCI and dementia.

Besides, it has been reported previously that SCD predicts a shorter time to progression compared to individuals without SCD [9, 30]. In our study, it was particularly stable SCD that was associated with a significantly shorter time to progression (median: 6.2 years). By contrast, unstable SCD revealed even a slightly longer progression-free survival time (median: 7.4 years) than controls (median: 7.2 years). However, we cannot exclude that individuals with stable SCD might potentially be more advanced in the pre-clinical phase of cognitive deterioration, thus, having a shorter time until MCI or dementia become overt. SCD might be experienced stable only after some subtle cognitive changes have occurred, perhaps allowing the chance to experience unstable SCD before such changes.

We have to consider some limitations. First, SCD was only measured by asking a simple question concerning memory problems. Even though the question “Do you have problems with your memory?” may be a valid measure for global memory functioning [31], there is a lack of psychometric data concerning its longitudinal use. Future investigations of the stability of SCD with more comprehensive assessments (e.g., assessing other domains but memory or asking for related worries) could shed more light on the potential link to MCI and dementia progression risk.

Second, current research criteria on SCD [3] also include biomarker abnormalities consistent with the AD pathology. In our study, we were not able to evaluate biomarkers in relation to patterns of SCD progression – future studies in this regard might be useful. Third, we otherwise applied those SCD research criteria which lead to the exclusion of a substantial proportion of the LEILA75+ participants. It is questionable whether such strict criteria that exclude major psychiatric, neurological or medical disorders reflect the actual at risk population for progression to MCI and dementia, especially as older adults can often simultaneously have such comorbidity.

Finally, the exclusion of such a substantial part of the cohort may also limit the generalizability of our results, even though the LEILA75+ study does have a population-based design. On the other hand, to the best of our knowledge, we are the first to present results on SCD patterns in community-dwelling elders.

## Conclusion

Our present results, though preliminary, suggest an increased risk of progression to MCI and dementia in individuals with stable SCD, i.e., consistent repeated SCD reports, compared to unstable SCD. Baseline cognitive scores, though within a normal range, seem to be a driver of progression in stable SCD. This may have implications for both research and clinical practice. In clinical practice, special attention should be put on repeated reports of SCD. Concerning research, further studies would be useful to establish whether stability, e.g., in form of a time criterion, could hold as an additional SCD research feature.

## Abbreviations

95 % CI: 95 % confidence interval
AD: Alzheimer’s disease
ADL: Activities of daily living
CASMIN: Comparative Analysis of Social Mobility in Industrial Nations
CDR: Clinical Dementia Rating Scale
CES-D: Center of Epidemiologic Studies Depression Scale
CON: Controls, i.e., individuals without SCD
HR: Hazard ratio
IADL: Instrumental activities of daily living
LEILA75+: Leipzig Longitudinal Study of the Aged
MCI: Mild cognitive impairment
MMSE: Mini Mental State Examination
SCD: Subjective cognitive decline
SIDAM: Structured Interview for the Diagnosis of Dementia of Alzheimer type, Multi-infarct Dementia and Dementia of other Aetiology according to DSM-III-R, DSM-IV, and ICD-10
